# Opinion: Increased calorie gain from lactose digestion could contribute to selection for lactase persistence

**DOI:** 10.1371/journal.pgen.1010612

**Published:** 2023-02-09

**Authors:** David Curtis

**Affiliations:** UCL Genetics Institute, University College London, London; University of Pennsylvania, UNITED STATES

## Summary

Lactase persistence refers to the retained ability of adults to express the gene for lactase and is recognised to be common in human populations which use domesticated animals. In some adults without lactase persistence drinking milk produces unpleasant abdominal symptoms, a condition termed lactose intolerance. Researchers studying the evolution of lactase persistence consistently take the view that selection pressures are mediated through lactose intolerance, meaning that the key issue is that lactase persistent individuals are able to consume milk without adverse consequences. However, most lactase non-persistent individuals are also able to freely consume milk. An alternative view is that lactase persistence considerably increases the calorific benefits of milk-drinking through allowing lactose to be used as an energy source and it is argued that this may be a key factor in driving selection for this trait.

Lactase is an enzyme present in the lining of the small intestine which breaks down lactose into glucose and galactose, which are readily absorbed. Although in most mammals lactase expression decreases markedly after infancy it has long been recognised that in human populations with domesticated animals it is common for lactase activity to continue into adulthood, a trait termed lactase persistence (LP) [1]. If lactase non-persistent (LNP) adults drink milk then the undigested lactose reaches the large bowel and people may experience abdominal symptoms such as bloating, flatulence and diarrhoea which mean that they are unable to drink significant quantities of milk, a condition termed lactose intolerance.

A number of theories have been put forward regarding the nature of the factors driving selection for LP as a trait which would allow adults to consume milk, allowing them to benefit from factors such as intake of calories, protein, vitamin D or simply pathogen-free fluid, [2]. A recently published study examined the relationship between LP and indicators of ancient milk usage, famine and pathogen exposure as well noting the lack of association of the LP genotype with milk consumption in a modern cohort [3]. The authors postulated that, in conditions of famine and/or exposure to pathogens, diarrhoeal disease mortality could have been increased in LNP individuals drinking milk, and that this mechanism could have driven LP selection.

Here, I argue that the exclusive focus on LP as allowing individuals to consume milk without adverse consequences overlooks a key consideration, which is that LP allows individuals to derive substantially increased nutritional benefit from milk when they do consume it because they are able to digest the lactose it contains. The literature around the molecular genetics of LP has developed in the context of understanding the main phenotype of relevance to modern, affluent populations, which is lactose intolerance, and the LP-conferring variant which was the subject of the recent study, rs4988235-A, was identified in a linkage and association study of adult-type hypolactasia [4]. The report of this study wrongly conflated the two concepts by referring to “lactase non-persistence (lactose intolerance)” as if they were equivalent whereas in fact, as Evershed and colleagues point out, most LNP individuals are not lactose intolerant and can drink milk without problems [3]. In reality, there is only a weak relationship between LNP and clinical symptoms attributed to lactose intolerance [5]. Nevertheless, it seems that the consensus became established that LNP exerted its effects through causing lactose intolerance so that LNP individuals would either consume less milk and be deprived of its benefits or else, as suggested in the recent study by Evershed and colleagues, would consume milk but have their health fatally compromised by symptoms such as diarrhoea.

In fact, there is no evidence that LNP status has any large influence on milk consumption nor that it has serious effects on health. In modern populations rs4988235-A is associated with obesity and with changes in the gut microbiome [6,7]. However, although lactose intolerance seems to be a somewhat unusual feature occurring in only a minority of LNP individuals, a consistent impact on all LNP individuals would be the failure to break down lactose and absorb the products, glucose and galactose, meaning that they would derive less calorific benefit from any milk which they did consume. The effect size is appreciable. The mass and energy content of the macronutrients in 1 litre of modern bovine milk consist of fat 33 g (297 cal), lactose 53 g (212 cal) and protein 32 g (128 cal) [8]. Taking just the fat and lactose to be sources of energy, this would mean that an LP individual could derive up to 70% (212/297) more calories from milk than an LNP individual. In evolutionary terms this could provide a significant advantage, perhaps especially in conditions of famine and/or high pathogen exposure, as were identified as promoting selection for LP in the recent study [3]. The energy advantage might be reduced to around half of this if colonic bacteria were able to ferment undigested lactose and if fermentation products could be absorbed to provide energy, but the difference would nevertheless be considerable [9].

Interestingly, researchers in the 1970s did propose that deriving increased calories from milk should be considered as a possible driver of selection for LP but their contribution is completely overlooked in the modern literature [10–12]. In more recent reports there are very many mentions of the calorific benefits of milk consumption but when one reads the sources closely these invariably turn out to refer to the fact that drinking milk in general provides calories, not that LP individuals derive more calories from milk than LNP individuals. I have been able to discover only one publication which refers to the specific calorific advantage which LP might bestow. In the middle of a long review article Ségurel and Bon state: "What is clear, however, is that LNP individuals cannot derive large amounts of glucose from any dairy products, as lactose, representing about 30% of the calories in human milk, is the sole sugar in milk." [13] However they then go on to immediately dismiss this as being of any relevance by citing three sources, none of which does in fact consider the specific calorie gains conferred by LP. Subsequently they state that LP individuals will derive approximately twice as many calories from lactose as LNP individuals but say that this difference is "not that high". Thus, while they do note that specific calorific benefits are conferred by LP they do not see this as potentially conferring an evolutionary advantage, with a consequence being that contemporary researchers fail to even consider this possibility [3].

In fact, the specific calorific benefits conferred by LP do appear to be substantial. It is recommended that in future studies investigating possible mechanisms of selection for LP-conferring variants researchers should consider the concomitant increased calorific value of milk as well as the possible adverse consequences of lactose intolerance.
